# A meta-analysis: neoadjuvant chemotherapy *versus* primary surgery in ovarian carcinoma FIGO stageIII and IV

**DOI:** 10.1186/1477-7819-11-267

**Published:** 2013-10-10

**Authors:** Ma Dai-yuan, Tan Bang-xian, Li Xian-fu, Zhou Ye-qin, Cai Hong-Wei

**Affiliations:** 1 Department of Oncology, the First Affiliated Hospital of North Sichuan Medical College, Nanchong 637000, People’s Republic of China

**Keywords:** Ovarian carcinoma, Primary surgery, Neoadjuvant chemotherapy

## Abstract

**Background:**

The purpose of the current study is to analyze the existing data comparing neoadjuvant chemotherapy with primary debulking surgery (PDS) in patients with advanced ovarian carcinoma.

**Methods:**

Patients with stage IIIC and IV ovarian cancer were identified from articles in Medline, PubMed, Cochrane Library, and EMBASE database (1989 to February 2013). Two authors independently extracted the data. To assess the risk of bias of included literatures, Cochrane Collaboration’s risk of bias tool was used. Meta-analysis on literatures was conducted by using RevMan 5.2 software.

**Results:**

Two high-quality randomized controlled trials (RCTs) met the inclusion criteria. These multicenter trials randomized 1,220 women with stage IIIc/IV ovarian cancer to NACT or PDS followed by chemotherapy. There were no significant differences between the study groups with regard to overall survival (OS) (1,120 women; HR 0.98; 95% CI 0.85 to 1.14) or progression-free survival (PFS) (1,120 women; HR 1.03; 95% CI 0.91 to 1.16).

**Conclusion:**

There was no statistical difference in median OS and PFS between the two treatment groups. With regard to selecting who will benefit from NACT, treatment should be tailored to the patient and should take into account respectability, age, histology, stage, and performance status.

## Review

### Introduction

An estimated 22,280 new cases of ovarian cancer are expected in the US in 2012 [1]. Ovarian cancer causes more deaths than any other cancer of the female reproductive system [1]. In most patients with ovarian carcinoma, the disease is diagnosed at an advanced stage and they usually have a very poor prognosis [2].

Primary surgical treatment of ovarian cancer has advantages in terms of diagnosis, staging, and tumor debulking [3,4]. The value of debulking surgery is well established in FIGO stage III epithelial ovarian cancer [5]. Most women will have widespread disease, therefore surgery alone does not cure the disease. Neoadjuvant chemotherapy (NACT) prior to surgical debulking proposes to increase the proportion of patients who may be optimally cytoreduced, while decreasing surgical morbidity and mortality [6].

Several retrospective studies have shown that there was no difference in overall survival (OS) or progression-free survival (PFS) for patients with advanced ovarian cancer treated with neoadjuvant chemotherapy compared with primary debulking surgery (PDS) [7-11]. However, the result of a meta-analysis of Bristow and Chi [12] involving 835 patients suggested that NACT, compared with PDS, was associated with a worse OS and it was suggested that the definitive operative intervention should be undertaken as early in the treatment program as possible. But a more recent meta-analysis [13] of multiply central randomized trials concluded that survival was similar in patients treated with NACT followed by interval debulking surgery compared to primary debulking followed by chemotherapy and criticized the meta-analysis of Bristow and Chi [12].

Since randomized controlled trials (RCTs) are the 'gold standard’ of evidence-based medical research, we hope that a review of randomized evidence may clarify what the benefits and risks are of using NACT for women with advanced ovarian cancer, compared with the standard treatment of PDS [13,14]. The purpose of the current study is to analyze the existing data comparing NACT with PDS in patients with advanced ovarian carcinoma which included more RCTs.

### Methods

#### Literature research

We undertook computerized literature searches of MEDLINE, PubMed, Cochrane Library, and EMBASE databases, from their inception to February 2013. Search terms were 'ovarian carcinoma’, 'ovarian cancer’, 'neoadjuvant chemotherapy’, and 'primary surgery’. These terms were used in different combinations with each other. Appropriate references cited by the retrieved studies were also identified.

#### Study selection

Publications were selected for initial review if the research subjects were patients with International Federation of Gynecology and Obstetrics (FIGO) stage III and IV ovarian cancer who underwent NACT and PDS. In order to fully exclude the possibility of selection bias, only RCTs of NACT *versus* PDS were permitted. Duplicate publications or data were carefully reviewed by two of the authors and the larger (primary decision) or the most recent publication was included.

Data on trial size, patient characteristics (age, sex, AF duration, left ventricular size, left ventricular ejection fraction, and so on), procedure duration, and patient number of sinus rhythm maintenance without anti-arrhythmic drugs were extracted. Included studies were reviewed based on randomization, allocation concealment, blinding, loss of follow-ups, and ITT. The truths of the studies were divided into three grades according to Cochrane system evaluation handbook: Grade A, cases met all evaluated standards and had correct methodology, which had low risk of bias; Grade B, cases did not describe one or several standards, which had moderate risk of bias; Grade C, cases had one or several standards incorrect, which had high risk of bias.

#### Data extraction

All the work of the literature search was independently reviewed by two authors to identify relevant trials that met the inclusion criteria and checked by an independent reviewer. Disparities were resolved by discussion. The following data were extracted from each study: (1) publication and trial characteristics, including the first author’s name, publication year, study period, center, study design, and sample size; (2) clinical data, including patient data (gender, age, quality of life) and tumor data (histological characteristics and grade classifications); (3) data of outcome, including value of OS and PFS. All data were centrally reanalyzed and checked for inconsistencies. In this article, the primary endpoint was OS. Secondary endpoints were PFS. OS was defined as the time from distribution until death from any cause or to the last follow-up that was used as a date of censoring. PFS was defined as the time from distribution to relapse or death from any cause, whichever came first. Data for patients alive without progression were censored on the date of last follow-up.

#### Statistical analysis

Meta-analyses were carried out by one author using RevMan5.2. For survival outcomes (OS and PFS), the log RRs from the various trials were combined in a meta-analysis using the Generic Inverse Variance facility of RevMan5.2. As a data analysis method, a fixed effect model was applied when the homogeneity between studies was verified. An I^2^ test was applied to determine between-study heterogeneity.

### Results

#### Search result

**Figure 1:**
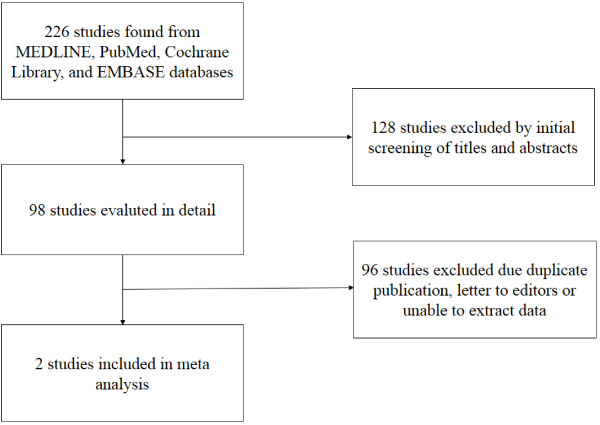
Process of study selection.

The search process of the study is shown in Figure 1. A total of 226 publications were identified after initial search. The titles and abstracts of these articles were examined to exclude irrelevant trials. We also examined the reference lists of all relevant letters, editorials, and review articles. As a result, two articles possibly met the selection criteria (Peter G Rose et al., 2004; IgnaceVergote et al., 2010). Subsequently, the full text of these studies was examined thoroughly.

#### Overall survival

**Figure 2:**
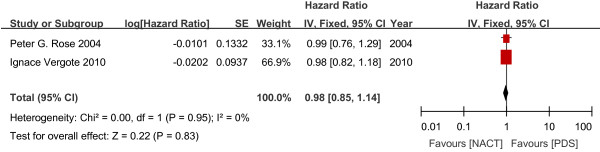
Overall survival outcome.

There was no significant difference in OS between the NACT and PDS groups (1,120 women; HR 0.98; 95% CI 0.85 to 1.14; Figure 2).

#### Progression-free survival

**Figure 3:**
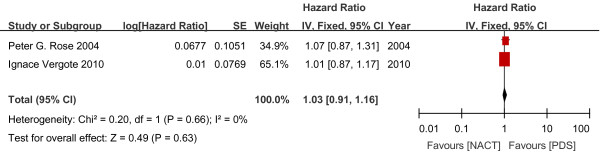
Progress-free survival outcome.

The existing data showed no significant difference in PFS between the NACT and PDS groups (670 women; HR 1.01; 95% CI 0.87 to 1.17; Figure 3).

## Conclusion

There was no statistical difference in median OS and PFS between the two treatment groups. With regard to selecting who will benefit from NACT, treatment should be tailored to the patient and should take into account respectability, age, histology, stage, and performance status.

### Discussion

The clinical basis of aggressive cytoreductive surgery in the initial management of ovarian cancer is the significantly improved survival gained by those patients in whom optimal cytoreductive surgery was accomplished [14]. The presence of residual disease after surgery is one of the most adverse prognostic factors for survival. Therefore, although the definition of optimal cytoreduction has been modified over the last two decades, it is generally agreed that every attempt should be made to surgically resect as much disease as safely possible [15]. There are many hypothesized advantages to NACT in women who have advanced ovarian cancer that likely cannot be optimally cytoreduced. Chemotherapy can resolve pleural effusions and ascites and improve the patient’s performance status prior to surgery [16,17]. There have been reports of subjective improvements in the sense of wellbeing and quality of life [18,19]. It can decrease tumor volume and increase respectability. Thus, patients may have less intraoperative blood loss, shorter operative times, less intensive care unit admissions, and shorter length of hospital stay [4,14,19]. These issues are particularly important for patients with medical co-morbidities and a low probability of cure.

Primary cytoreductive surgery is still the gold standard in the treatment of ovarian carcinoma [20]. NACT for advanced unresectable ovarian carcinoma led to the selection of a group of patients sensitive to chemotherapy, in whom secondary cytoreductive surgery can be achieved in a less aggressive manner. Also NACT prevents mutilating surgery in patient with a very poor prognosis either because of progressive disease or because of primary chemoresistance. These findings must be confirmed by a larger prospective study [21].

There are several limitations to this current study that must be considered in interpreting the data. This is a retrospective study, the limitations imposed by these attributes have to be borne in mind when interpreting or using the findings [22]. A standardized protocol to monitor chemotherapy response was not used in these patients thus potentially introducing lead-time bias, nor was our study designed to control for various second and further-line treatment regimens, which may have the ultimate impact on OS [23].

## Competing interests

The authors declare that they have no competing interests.

## Authors’ contributions

DM independently searched references and took charge of data statistics and drafted the manuscript. BT and XL searched references and extracted the parameters from each study. YZ and HC participated in the manuscript revision. All authors read and approved the final manuscript.
